# *Helicobacter pylori* Efflux Pumps: A Double-Edged Sword in Antibiotic Resistance and Biofilm Formation

**DOI:** 10.3390/ijms252212222

**Published:** 2024-11-14

**Authors:** Paweł Krzyżek

**Affiliations:** Department of Microbiology, Faculty of Medicine, Wroclaw Medical University, 50-368 Wroclaw, Poland; pawel.krzyzek@umw.edu.pl

**Keywords:** *Helicobacter pylori*, efflux pumps, efflux systems, transporters, antibiotic tolerance, antibiotic resistance, biofilm

## Abstract

*Helicobacter pylori* is a major pathogen associated with various gastric diseases. Despite decades of research, the treatment of *H. pylori* remains challenging. One of the primary mechanisms contributing to failures of therapies targeting this bacterium is genetic mutations in drug target sites, although the growing body of scientific data highlights that efflux pumps may also take part in this process. Efflux pumps are proteinaceous transporters actively expelling antimicrobial agents from the interior of the targeted cells and reducing the intracellular concentration of these compounds. Considering that efflux pumps contribute to both antimicrobial resistance and biofilm formation, an in-depth understanding of their properties may constitute a cornerstone in the development of novel therapeutics against *H. pylori*. In line with this, the aim of the current review is to describe the multitude of efflux pumps produced by *H. pylori* and present the data describing the involvement of these proteins in tolerance and/or resistance to various classes of antimicrobial substances.

## 1. Introduction

*Helicobacter pylori* is a Gram-negative, flagellated, microaerophilic bacterium that colonizes the human stomach and is a leading etiological agent of gastrointestinal pathologies [1]. Despite over 40 years of research on the physiology and pathogenicity of this microorganism, infections caused by this bacterium are still among the most common in the world [2]. *H. pylori* plays a key role in the development of chronic progressive gastritis and various gastrointestinal diseases, including gastric/duodenal ulcers and stomach neoplasms (gastric adenocarcinomas and gastric mucosa-associated lymphoid tissue lymphomas) [3,4]. Most infections caused by this bacterium are acquired in childhood and develop into chronic lifelong diseases if not effectively treated [1,5]. The progression of the infection caused by *H. pylori* depends on many factors, including the virulence of the strain, the genetic background and efficiency of the host’s immune system, as well as the dietary habits and medical history of the patient [6,7].

Shortly after the first successful cultivation of *H. pylori* and the discovery of its role in the development of gastric diseases in the 1980s, the high antibiotic sensitivity of this microorganism was reported. Unfortunately, the discovery of *H. pylori* and the first attempts to search for effective therapies to combat it were accompanied by the global trend of the antibiotic resistance spreading [8]. It turned out quite quickly that the use of a single antibiotic only reduces the amount of this microorganism in the stomach, while the achievement of the complete eradication is practically impossible [9]. These therapeutic difficulties are largely a result of specific physicochemical conditions in the gastric environment, including very low pH, a thick layer of mucin, and poor accessibility to blood-borne antibiotics and immune cells [9,10]. Despite the importance of the above factors, antibiotic resistance is indicated as the main determinant influencing the possibility of curing the infection caused by *H. pylori* [11,12]. Chromosomally encoded point mutations in drug target sites represent the most explored mechanism of antibiotic resistance of *H. pylori* and include modifications of the following genes: *pbp1a*, *pbp2* or *pbp3* in amoxicillin resistance, *23S rRNA* in clarithromycin resistance, *16S rRNA* in tetracycline resistance, *rpoB* in rifabutin resistance, *gyrA* or *gyrB* in levofloxacin resistance, and *rdxA* and/or *frxA* in metronidazole resistance (Table 1) [11,13].

The widespread overuse and inappropriate assignment of antibiotics, largely in the treatment of respiratory tract infections, genital tract infections and dental infections, has cast a shadow on the possibility of an effective *H. pylori* eradication [14,15]. This issue has become particularly important, as over the last two decades a gradual increase in antibiotic resistance of this bacterium has been observed [16,17]. As shown in a very detailed meta-analysis of 66142 *H. pylori* isolates from 65 countries around the world, both primary (treatment-naïve) and secondary (treatment-exposed) resistance to metronidazole, clarithromycin and levofloxacin of the examined *H. pylori* strains exceeded the alarming level of >15% in almost all monitored areas [18]. In this regard, the lowest level of resistance was recorded in the Americas and Europe, while the highest was in Africa and the Eastern Mediterranean region. It is also worth mentioning that patients undergoing unsuccessful therapies had a higher prevalence of resistant strains than treatment-naïve ones. For example, in Europe, these differences were equal to 48% vs. 18%, 65% vs. 56% and 30% vs. 19% for clarithromycin, metronidazole and levofloxacin, respectively.

To counteract the widespread antibiotic resistance and therapeutic problems, a combination of three (triple therapy) or four (quadruple therapy) drugs is currently recommended in the *H. pylori* treatment. These include proton pump inhibitors and a mixture of several antimicrobial agents (amoxicillin, clarithromycin, metronidazole, levofloxacin, tetracycline, rifabutin, or bismuth salts) [19]. The need to use multi-component therapy against *H. pylori* has resulted in the increased prevalence of multidrug resistance (MDR), a process defined as simultaneous resistance to at least three groups antibiotics at the same time [20,21]. While the resistance to single antibiotics in *H. pylori* is caused by chromosomal mutations in drug target sites, the phenomenon of MDR is more complex and is often caused by intricate physiological changes, including a morphological transformation into coccoid forms, the development of biofilm, and an increased activity of efflux pumps [11,12]. The importance of a transition into coccoid forms and biofilm formation in the pathogenesis of *H. pylori* is relatively often discussed by the scientific community, including reviews broadly describing these phenomena [22,23,24,25,26]. A topic that still remains poorly explored, while potentially playing a vital role in the propagation of MDR among *H. pylori*, is the activity of efflux pumps.

Therefore, the aim of the current review is to describe the multitude of efflux pumps produced by *H. pylori* and to present original data from the last twenty-five years describing the involvement of these structures in tolerance and/or resistance to various classes of antimicrobial substances.

## 2. Characteristics of Efflux Pumps

An extracellular release of potentially harmful compounds is one of the basic mechanisms by which microorganisms cope with the environmental stress [27,28,29,30]. This applies to both Gram-negative and Gram-positive bacteria, as well as eukaryotic cells. This activity involves efflux pumps, which are defined as proteinaceous cytoplasmic transporters involved in the translocation of chemical compounds across the membrane. The primary function of efflux pumps is the extracellular removal of various substances that may disturb the functioning of cells, including antibiotics, organic compounds, dyes, detergents, and heavy metal ions. However, it should be taken into account that efflux pumps function not only as transporters of harmful substances, but also as regulators of microbial physiology by participating in the secretion of virulence factors or autoinducers (compounds involved in the intercellular communication) [27,31].

Based on the protein homology sequence, the spatial structure and the energy source, efflux pumps can be divided into six superfamilies: ATP-binding cassette (ABC), multidrug and toxic compound extrusion (MATE), small multidrug resistance (SMR), major facilitator superfamily (MFS), resistance nodulation cell division (RND), and proteobacterial antimicrobial compound efflux (PACE) [27,28,29,30]. One difference between these superfamilies is the source of the energy used to eject substances to the extracellular space. Those belonging to the ABC superfamily are classified as primary efflux pumps, as they obtain the energy for their activity from the ATP hydrolysis. The remaining five superfamilies of pumps are classified as secondary and acquire the energy for their functioning from the electrochemical gradient generated by protons or other ions like Na^+^ (the proton or sodium motive force) [27,28]. Although the variety of efflux pumps exist, it is estimated that each of them has a relatively low substrate specificity. This translates into a situation during which a specific efflux pump can export different types of compounds, while a given type of substance can be transported simultaneously by several different efflux pumps [28,30]. Therefore, a low substrate selectivity of efflux pumps correlates with a high level of tolerance to many classes of antimicrobial substances and constitutes an evitable risk of MDR development [28,32].

**Figure 1:**
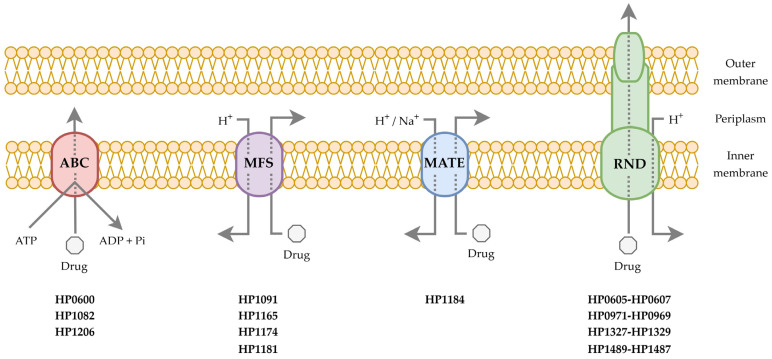
A diagrammatic representation of four superfamilies of efflux pumps present in *Helicobacter pylori*. Representatives of each superfamily are provided below the scheme.

As the literature review of studies shows, only four superfamilies of efflux pumps have been identified so far in *H. pylori*: RND, MFS, ABC, and MATE [33,34] (Figure 1). Their detailed characteristics and description of known functions are presented below.

## 3. Involvement of Efflux Pumps in Resistance to Antimicrobial Compounds

### 3.1. The RND Superfamily

The RND superfamily of efflux pumps is composed of 12 transmembrane helices, which are separated by two large loops forming asymmetric trimers and outer loops which are equipped with ligand binding sites [27,28,29,30]. The transmembrane domain operates usually as a channel for protons to obtain the energy needed for the translocation of substrates. The RND pump is assembled from an outer membrane factor, a periplasmic adapter protein and an inner membrane transporter, creating all together a three-component system. Because of this tripartite nature, RND efflux pumps are larger than other bacterial efflux proteins. It is indicated that the RND superfamily is of particular importance in generating resistance in Gram-negative bacteria. Indeed, a review of the data shows that this statement is also true for *H. pylori* [33,34]. Four tripartite RND systems have been identified in this bacterium, including HefABC (HP0605-HP0607), HefDEF (HP0971-HP0969), HefGHI (HP1327-HP1329) and HP1489-HP1487 (Table 2 and Figure 1).

#### 3.1.1. HefABC (HP0605-HP0607)

Among all four RND representatives, the HefABC pump is definitely of the greatest interest to scientists. Comparative studies of antibiotic-sensitive and MDR *H. pylori* strains performed by several independent groups indicated that the expression of *hefABC* is several times higher in antibiotic-resistant strains [35,36,37,38]. This pump is characterized by a very low substrate selectivity, which was demonstrated by an increase in the sensitivity of the tested *H. pylori* strains to several different antimicrobial compounds simultaneously when using both efflux pump inhibitors [37] or deletants of the genes encoding this pump [36,39]. The use of efflux pump inhibitors contributed to a decrease in minimal inhibitory concentrations (MICs) of five out of the nine antibiotics assessed (cefotaxime, ceftriaxone, erythromycin, tetracycline and chloramphenicol) [37]. These observations are consistent with the sensitivity profile of ∆*hefA* mutants, for which a decline of MICs for cefotaxime, clarithromycin, gentamicin and chloramphenicol was noticed [36]. For ∆*hefC* mutants, the range of antibiotics with reduced MICs was slightly wider, although it most likely resulted from an extended spectrum of tested substances (as many as 20) [39]. These deletants showed an increased susceptibility to penicillins, cephalosporins, novobiocin, clindamycin, erythromycin, tetracycline and the fluorescent dye ethidium bromide. 

Research carried out in later years, unlike the earlier ones determining the role of HefABC in resistance to many different groups of antimicrobial compounds, focused on verifying the importance of this efflux pump in resistance to specific antibiotics routinely prescribed for the *H. pylori* treatment. In this context, much attention has been paid to the involvement of HefABC in metronidazole resistance. This interest most likely started in 2005 from the observations made in the first research article focusing on *H. pylori* efflux pumps, in which an increase in the sensitivity to metronidazole was observed in double ∆*hefA*∆*hefD* deletants [40]. Later studies showed that the higher expression of *hefA* was observed in clinical metronidazole-resistant strains, suggesting that the activity of this pump may play an important role in response to metronidazole exposure and the acquisition of resistance to this antibiotic [41,42,43]. Indeed, with the use of Δ*hefA* deletants, it was demonstrated that the formation of mutations in *rdxA*, a gene classically associated with metronidazole resistance, was slower than in wild strains [38]. Moreover, the introduction of the ∆*hefA* deletion to metronidazole-resistant strains reversed already established resistance to this antibiotic [38,43]. Although the vast majority of studies focused on the participation of the HefABC pump in the development of resistance to metronidazole, other reports indicated the involvement of this tripartite system in the propagation of resistance to clarithromycin [38,44] and amoxicillin [45]. In addition to the above-presented experiments on antibiotics, studies of Van Amsterdam et al. [40] and Trainor et al. [46] also examined the role of HefABC in resistance to bile salts and ceragenins (cationic bile salt derivatives that mimic the activity of endogenous antimicrobial peptides). It was confirmed that HefABC is involved in the response to these compounds and an increase in tolerance to them may occur, e.g., through the transport of cholesterol molecules and cholesterol-dependent changes in the bacterial cell membrane fluidity.

#### 3.1.2. HefDEF (HP0971-HP0969)

The second best-studied representative of RND proteins of *H. pylori* is the HefDEF system. One of the first studies on *H. pylori* efflux pumps, published in 2006, showed the strong involvement of this pump in the specific ejection of heavy metal ions and generation of tolerance to their presence [47]. Moreover, it was observed that this operon was crucial for the urease activity and therefore stomach colonization of Mongolian gerbils. It is suggested that HefDEF takes part in the extracellular export of Cd^2+^ and Zn^2+^ ions in the acid environment in order to maintain the necessary level of Ni^2+^ and the proper activity of urease of *H. pylori*. At the same time, this research team did not detect any changes in the antibiotic sensitivity profile in mutants of this pump. Interestingly, reports from subsequent years questioned the observations regarding the lack of an impact of HefDEF in antibiotic resistance, which was most likely the result of differences in the methodology for assessing these properties (cultures on solid vs. liquid media). Several independent studies have suggested that this pump is involved in the development of resistance to metronidazole [38,40,41], clarithromycin [38,48] and levofloxacin [49]. Additionally, similar to *hefABC*, the expression of *hefDEF* was higher in MDR strains than in susceptible ones [38].

#### 3.1.3. HefGHI (HP1327-HP1329)

Another representative of the RND superfamily in *H. pylori* is the HefGHI pump. Based on RNA profiling of the reference *H. pylori* strain, it was found that this system, together with the secreted protein HP1326 (CrdA), is involved in maintaining cytoplasmic copper homeostasis and tolerance to this metal ions [50]. So far, the activity of this pump has been associated with the acquisition of resistance to levofloxacin [49].

#### 3.1.4. HP1489-HP1487

The last representative of the RND superfamily in *H. pylori* is the HP1489-HP1487 pump. For a very long time, little was known about the function of this tripartite system. The only evidence showing any biological function of this pump was coming from the observation indicating an increased sensitivity of Δ*hp1489* mutants to ethidium bromide [40]. New light on the function of these proteins was shed by a recently published study highlighting the involvement of HP1489-HP1487 in the motility of *H. pylori* [51]. The experiments proved that deletants of genes encoding this efflux pump had a reduced number of flagella per single bacterial cell, suggesting the involvement of this system in the flagellar motor stabilization in *H. pylori*. Currently, the direct cause of this phenomenon is unknown, but the authors implied that this system may participate in the extracellular secretion of substances destabilizing flagella or may take part in the delivery of flagella-stabilizing compounds to their close proximity.

### 3.2. The MFS Superfamily

The MFS superfamily of efflux pumps is the largest known group of secondary active transporters found in every domain of life [27,28,29,30]. These proteins utilize the energy from the proton motive force to transport substrates across the membrane. Primarily, this superfamily consists of uniporters (a movement of a solute independently of ions), antiporters (a movement of an ion and a solute in opposite directions) and symporters (a movement of a solute and an ion in the same direction). The members of this superfamily of efflux pumps perform their function mainly as monomers made of 12 or 14 transmembrane segments organized into two domains. In *H. pylori*, the literature data describing the biological activity of pumps representing this superfamily include HP1165 (TetA), HP1174 (GluP) and HP1181. It is also indicated that HP1091 (KgtP) is a member of this superfamily, but its function is not known yet (Table 2 and Figure 1).

#### 3.2.1. HP1174 (GluP)

The best characterized representative of the MFS superfamily in *H. pylori* is undoubtedly HP1174 (GluP), which is a Na^+^-dependent glucose and galactose transporter [52]. Regardless of the role of this protein in the transport of hexoses, this pump may also play an important role in generating resistance to various antibiotic classes. In a comparative study of MDR and antibiotic-sensitive strains, the former presented a 7-fold higher gene expression of this pump [53]. The ∆*gluP* deletants showed a more than 3-fold increase in the accumulation of the fluorescent dye Hoechst 33342 compared to the wild strain. Additionally, the mutation of *gluP* sensitized bacteria to a wide range of antibiotics, including penicillins (amoxicillin, ampicillin and penicillin), metronidazole, tetracycline and furazolidone, but not ciprofloxacin or clarithromycin. Recently published study discovered that the activity of GluP is controlled by the oxidative stress-responsive regulator HP1021 [54]. Through HP1021, *H. pylori* controls GluP-dependent glucose transport and maintains the energy balance of bacterial cells.

#### 3.2.2. HP1165 (TetA)

Another representative of the MFS superfamily in *H. pylori* is HP1165 (TetA). The team of Li et al. [55] found that this protein has almost 50% homology with TetA of *Clostridium perfringens*. Additionally, hypersensitivity to tetracycline and a 10-fold reduction in the MIC of this antibiotic was observed in ∆*tetA* deletants. It was noticed that in the absence of any point mutations in *16S rRNA*, a gene classically involved in resistance to TET, this pump can participate in the generation of intermediate-level resistance to this antibiotic (MIC = 4–6 µg/mL).

#### 3.2.3. HP1181

The last pump with a described function belonging to the *H. pylori* MFS superfamily is HP1181. A study on clinical strains of this bacterium demonstrated the involvement of this protein in resistance to ethidium bromide, penicillins, metronidazole, tetracycline and ciprofloxacin [56]. Additionally, a comparative analysis of a large pool of clinical *H. pylori* strains indicated that *hp1181* is present only in a minority of strains, while its presence is significantly higher in metronidazole- and levofloxacin-resistant strains [33].

### 3.3. The ABC Superfamily

The ABC superfamily is classified as primary efflux pumps, as the energy to translocate solutes is driven by the hydrolysis of ATP [27,28,29,30]. The ABC efflux pumps possess functional diversity, although all representatives share a basic common architecture consisting of two membrane-integral part domains transversing the membrane six times each (12 transmembrane domains) and two nucleotide-binding domains. Their transmembrane domains are involved in the binding of substrates, while nucleotide-binding domains are involved in hydrolyzing ATP molecules to obtain the energy for the transport. A review of the data analyzing the biological activity of pumps representing this superfamily in *H. pylori* indicates only two representatives, i.e., HP0600 (SpaB) and HP1082 (MsbA). It is demonstrated that HP1206 (HetA) is another example of a pump belonging to the ABC superfamily, but its function has not been known yet (Table 2 and Figure 1).

#### 3.3.1. HP1082 (MsbA)

A review article describing the structure and function of the MsbA protein showcases its common occurrence in various microorganisms and its involvement in the transport of lipids and hydrophobic drugs [57]. MsbA is a lipid flippase that transfers lipopolysaccharide components from the inner to the outer leaflet of the cytoplasmic membrane. A similar activity was also confirmed empirically for *H. pylori* [58]. It was noticed that the expression of *msbA* correlated with resistance of *H. pylori* to glutaraldehyde, an organic compound classically used to preserve biological samples. The ∆*msbA* mutants showed not only an increased sensitivity to glutaraldehyde, but also to the examined hydrophobic drugs (erythromycin, novobiocin and rifampicin). Moreover, a decrease in the amount of lipopolysaccharide was observed in these deletants. The above data indicate the pleiotropic effect of MsbA in *H. pylori* and the involvement of this protein not only in the transport of outer membrane components of this bacterium, but also non-specific resistance to various hydrophobic compounds with the antimicrobial activity. The participation of MsbA of *H. pylori* in resistance to antimicrobials was also proved by another study [59], in which the higher level of *msbA* expression was detected in MDR strains. Additionally, it was noticed that ∆*msbA* mutants were more sensitive to clarithromycin, chloramphenicol, ofloxacin and rifampicin.

#### 3.3.2. HP0600 (SpaB)

The second discussed representative of the ABC superfamily in *H. pylori* is SpaB. In a very detailed next-generation sequencing-based study performed on 112 clinical *H. pylori* strains, it was found that, in contrast to other analyzed efflux pump genes, *spaB* was present in a minority of them [33]. Nevertheless, it was observed that the proportion of strains possessing *spaB* was higher in metronidazole-resistant or levofloxacin-resistant strains, suggesting the involvement of this pump in the generation of antibiotic resistance of *H. pylori*. In line with the above data, in the another study, the higher expression of *spaB* was seen in MDR strains [59]. Moreover, deletion of *spaB* lead to an increased sensitivity of the tested strains to clarithromycin, chloramphenicol, ofloxacin and rifampicin.

### 3.4. The MATE Superfamily

The MATE superfamily is classified into the multidrug/oligosaccharide-lipid/polysaccharide flippase superfamily [27,28,29,30]. These transporters are composed of 12 transmembrane helices. The bacterial MATE transporters use a transmembrane H^+^ and Na^+^ gradient to drive the efflux of polyaromatic and cationic drugs. This is the least characterized superfamily of *H. pylori* efflux pumps, as so far the only representative with a described biological function is HP1184 (Table 2 and Figure 1). 

The data on the participation of HP1184 in *H. pylori* antimicrobial resistance come from only two research articles [40,56]. The first one showed that ∆*hp1184* mutants are characterized by a higher sensitivity to ethidium bromide [40]. The second study suggested the involvement of this pump in resistance to ethidium bromide, penicillins, metronidazole, tetracycline and ciprofloxacin [56].

**Table 2:** Efflux pumps of *Helicobacter pylori* and their involvement in antibiotic resistance.

Superfamily	Efflux Pump	Substrates	Association with MDR	Additional Biological Activities	References
RND	HP0605-HP0607 (HefABC)	penicillin G, piperacillin, amoxicillin, ceftriaxone, cefotaxime, gentamycin, tetracycline, erythromycin, clarithromycin, clindamycin, novobiocin, metronidazole, bile salts, ceragenins, ethidium bromide	+	Transport of cholesterol molecules and cholesterol-dependent changes in the cell membrane fluidity	[33,34,35,36,37,38,39,40,46]
	HP0971-HP0969 (HefDEF)	heavy metal ions (Cd^2+^, Zn^2+^, Ni^2+^), clarithromycin, metronidazole, levofloxacin	+	Modulation of urease activity and a role in the gastric colonization	[33,34,38,40,41,47,48,49]
	HP1327-HP1329 (HefGHI)	heavy metal ions (Cu^2+^), levofloxacin	*ND*	*ND*	[33,34,49,50]
	HP1489-HP1487	ethidium bromide	*ND*	Stabilization of the flagellar motor and a role in the motility	[33,34,40,51]
MFS	HP1091 (KgtP)	*ND*	*ND*	*ND*	[33,34]
	HP1165 (TetA)	tetracycline	*ND*	*ND*	[33,34,55]
	HP1174 (GluP)	amoxicillin, ampicillin, piperacillin, tetracycline, metronidazole, furazolidone	+	Transport of simple sugars and a role in the maintenance of the energy balance	[34,52,53,54]
	HP1181	amoxicillin, ampicillin, tetracycline, metronidazole, ciprofloxacin, ethidium bromide, levofloxacin	*ND*	*ND*	[33,34,56]
ABC	HP0600 (SpaB)	clarithromycin, chloramphenicol, rifampicin, ofloxacin, levofloxacin, metronidazole	+	*ND*	[33,34,59]
	HP1082 (MsbA)	clarithromycin, erythromycin, chloramphenicol, rifampicin, ofloxacin, novobiocin, glutaraldehyde	+	Transport and biogenesis of lipopolysaccharide	[33,34,58,59]
	HP1206 (HetA)	*ND*	*ND*	*ND*	[33,34]
MATE	HP1184	amoxicillin, ampicillin, tetracycline, metronidazole, ciprofloxacin, ethidium bromide	*ND*	*ND*	[34,40,56]

Abbreviations: MDR, multidrug resistant; *ND*, no data.

## 4. Involvement of Metabolic Transporters in Resistance to Antimicrobial Compounds

Diverse intracellular metabolites can be externalized [60,61,62]. Externalized metabolites are transiently available for other cells in the local environment and may therefore affect their physiological activities. In most cases, communally valuable metabolites are externalized via facilitated and active transporters in order to maintain appropriate metabolite levels or to carry out roles that differ from those in the cytoplasm. Transporters facilitating this process may often share many similarities with efflux pumps, while not necessarily fall within the classical categorization established for them. In *H. pylori*, several metabolic transporters have also been identified. Their primary function is associated with the transport of nutrients or substances related to the metabolism, but as the literature data show, they may also participate in generating resistance to some antibiotics (Table 3). 

The research by Geng et al. [63] focused on the analysis of the involvement of a wide range of metabolic transporters in generating resistance to clarithromycin in *H. pylori*. Among the examined transporters, four of them showed a significant increase in the expression after exposure to clarithromycin: HP0471 (KefB, potassium ion transport), HP0497 (sodium and chlorine ion transport), HP0939 (YckJ, amino acid transport) and HP1017 (RocE, amino acid transport). It was proved that the average expression of genes encoding these four transporters was significantly higher in clinical strains with clarithromycin resistance than in susceptible ones. Additionally, it was observed that the deletion of ∆*hp0471*, ∆*hp0497* or ∆*hp0939* in clarithromycin-resistant strains was accompanied by a reduction or a complete loss of resistance to this antibiotic. In another original article by this team [64], the authors decided to determine whether HP0471, HP0497 and HP0939 transporters may also impact resistance to other antibiotics. It was discovered that exposure to metronidazole or amoxicillin was associated with an increase in the expression of genes of all three tested transporters. Using the ∆*hp0471*, ∆*hp0497* and ∆*hp0939* deletants, an increased sensitivity to a number of chemically unrelated antibiotics, including penicillins, clarithromycin, tetracycline, metronidazole, ciprofloxacin and furazolidone, was demonstrated. On the other hand, the opposite effect was observed in strains overexpressing these genes. Finally, in a comparative analysis of clinical MDR and antibiotic-sensitive strains, the former showed a significantly higher expression of all three genes of these transporters. 

The above observations shed new light on the correlation between the microbial metabolism and tolerance/resistance to antimicrobial substances. As proved, this phenomenon does not have to be related solely to a reduction in the metabolic activity and formation of persisters [65,66], but it may also result from the increased activity of metabolic transporters, as determined in *H. pylori* [63,64] (Table 3).

## 5. Participation of Efflux Pumps in Biofilm Formation

Biofilm is a multicellular structure composed of microorganisms encased in an extracellular matrix [67,68]. A recent meta-analysis estimated that approximately 40–80% of cells on Earth live in the biofilm form, while only a minority in the planktonic phase [69]. For this reason, the medical and economic significance of the biofilm is of a paramount importance [70]. Taking into consideration the prevalence of biofilms and their potentially negative impact on the human health, the European Society of Clinical Microbiology and Infectious Diseases developed recommendations for the scientific community and health care providers to improve the diagnosis and treatment of the most common biofilm-associated infections [71]. These infections are characterized by a chronic nature and difficulties in the treatment, which is related to a number of unique properties of the biofilm, such as the intensification of genetic information exchange (the so-called horizontal gene transfer), the presence of a thick layer of extracellular matrix limiting the diffusion of antimicrobial compounds, as well as the presence of multiple microbial subpopulations with various phenotypic features (including those with a very limited metabolism and high tolerance to various antimicrobials—referred to as persisters) [67,68,72]. 

Especially in the last decade, the topic of biofilm formation by *H. pylori* and its importance in therapeutic difficulties have begun to gain momentum [22,23,24]. Several articles highlighted that the development of the biofilm by *H. pylori* is accompanied by an increase in tolerance to various groups of classically used antibiotics [73,74,75]. Interestingly, a correlation between genetically encoded resistance to certain antibiotics and biofilm formation properties of clinical *H. pylori* strains was also observed [76,77,78]. In relation to the above data indicating the great clinical importance of biofilm production by *H. pylori*, it seems reasonable to determine whether this process might be facilitated by the activity of efflux pumps. 

**Figure 2:**
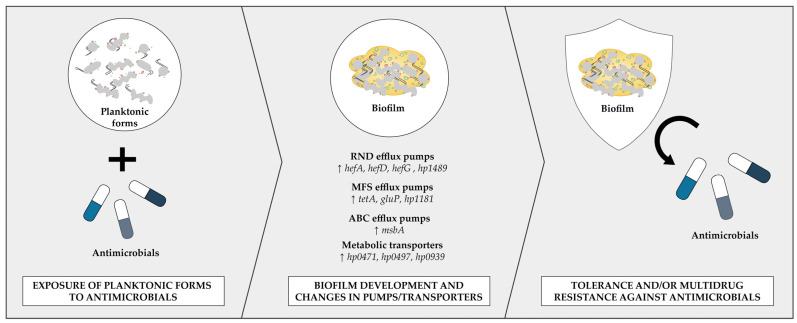
A schematic drawing showing a response of *H. pylori* to the antimicrobial exposure. This adaptative response is associated with changes in the expression profile of genes encoding efflux pumps and metabolic transporters during the planktonic-to-biofilm transition of *Helicobacter pylori*, which as a result lead to tolerance and/or multidrug resistance against antimicrobials.

Indeed, several studies attempted to determine the relationship between the expression of genes encoding efflux pumps or metabolic transporters and the transition of *H. pylori* from the planktonic to the biofilm phase [53,64,73,79,80] (Figure 2). The first article on this topic was published in 2013 [80]. The experiments were performed on the clinical *H. pylori* TK1402 strain, whose transition to the biofilm form was accompanied by a 2–4-fold increase in the expression of RND pump genes (*hefA*, *hefD*, *hefG* and *hp1489*). The team obtained similar results a few years later when determining the expression of these genes in the another clinical *H. pylori* strain—TK1049 [73]. In this case, compared to planktonic forms, the biofilm of this bacterium was characterized by a 2-fold increase in the expression of *hefA* and *hefD*, as well as a 6–7-fold increase in the expression of *hefG* and *hp1489*. Another research group conducted their analysis on three clinical *H. pylori* strains and found that the biofilm had several times higher expression of the RND pump gene, *hefA*, but also one from the MFS superfamily, *tetA* [79]. Another research team concentrated their experiments on the reference *H. pylori* 26695 strain, and decided to significantly expand the range of assessed efflux pump genes [53]. In that instance, scientists managed to demonstrate that the biofilm of this bacterium is characterized by a more than 2-fold increase in the expression of a gene from the ABC superfamily, *msbA*, as well as several representatives of the MFS superfamily: *tetA*, *gluP* and *hp1181*. In the last article describing this phenomenon, the research was also carried out on the reference *H. pylori* 26695 strain, although this time the focal point was placed on metabolic transporter genes: *hp0471*, *hp0497* and *hp0939* [64]. It was discovered that biofilm forms, compared to planktonic ones, had several to dozen times higher expression of genes encoding these transporters. Additionally, deletion of any of these genes led to a significant decrease in the biofilm production ability of the tested *H. pylori* strain.

These reports determining the relationship between the activity of efflux pumps and biofilm formation by *H. pylori*, although still very scare, may help in the future design of new therapies aimed at reducing antibiotic resistance and biofilm development of this pathogen. This type of approach was described in the review by Ren et al. [81], who discussed in details the possibility of inhibiting the biofilm development by using efflux pump inhibitors in various groups of microorganisms.

## 6. Inhibition of Efflux Pumps as a New Therapeutic Path

Taking into account the importance of efflux systems in mediating both antibiotic resistance and biofilm formation of *H. pylori*, it is worthwhile considering efflux pump inhibitors as a new avenue to fight this pathogen. Efflux pump inhibitors are typically considered as molecules that may specifically or non-specifically inhibit the activity of efflux systems, resulting in the inactivation of the drug transport or other pathogenicity traits, such as the biofilm development [81,82,83]. Since this activity can finally lead to the accumulation of antimicrobials inside the targeted cells, efflux pump inhibitors can be also applied as adjuvants of classical therapies. 

The usefulness of efflux pump inhibitors in enhancing the activity of antibiotics against *H. pylori* was noticed many years ago, although it had been narrowed to laboratory experiments aimed at showing the connection between the activity of efflux pumps and the generation of resistance in *H. pylori* [37,42,45,56,84,85,86]. Recent years have begun to bring new insights into their possible therapeutic applications. The first study, published just in 2021, demonstrated the ability of patchouli alcohol to reduce the expression of three RND pump genes (*hefA*, *hefG* and *hp1489*) in a concentration-dependent manner [87]. Another study tested the effect of Jinghua Weikang capsule on a number of phenotypic features of the reference *H. pylori* strain with induced resistance to metronidazole (*H. pylori* 26695-16R) [88]. It was found that this medication not only limited the expression of all four representatives of RND pump genes, but also reduced the amount of the biofilm formed by this bacterium. The next two articles, both published in 2024, also managed to demonstrate the ability of the tested preparations to inhibit both the expression of efflux pump genes and the capacity of *H. pylori* to form the biofilm [89,90]. One article showed that Qingre Huashi decoction reduced the expression of *gluP* and four RND pump genes [89], while the other proved the ability of *Mass Galla chinensis et camelliae Fermentata* to decrease the expression of *hefC*, *hefI*, *gluP* and three metabolic transporter genes (*hp0471*, *hp0497* and *hp0939*) [90]. 

The above data undoubtedly constitute a great encouragement to consider efflux pump inhibitors as a new mode to fight antibiotic-resistant strains of *H. pylori*. One of the most crucial features of an effective antimicrobial drug is its ability to interfere with vital functions of microbial cells [91,92]. Decades of studies on efflux pumps have proved their definitive role in antimicrobial tolerance and resistance, while the participation in microbial physiology is far less understood [31]. It is broadly documented that microbial drug-ejecting pumps are chromosomally encoded and therefore are considered to be intrinsic elements of the microbial genome [27,31,93]. The polyspecifity and the high degree of conservation of efflux pumps strongly implies that the ability of these proteinaceous structures to facilitate antimicrobial resistance and tolerance is a fortuitous by-product of ancient physiological functions [93]. Although the topic of the evolutionary history of *H. pylori* efflux pumps is currently in its infancy, one of the recent studies examined a wide pool of clinical strains of this bacterium and showed that among the 18 efflux pump genes tested, 16 of them were present in almost 100% of the strains [33]. This confirms the above statement about the high conservation of efflux pumps and creates the opportunity for using efflux pumps as drug targets in the future. Certainly, however, detailed pharmacological and toxicological studies of efflux pump inhibitors are necessary to realistically take into account the possibility of introducing these compounds as new preparations in the treatment of patients infected with *H. pylori*.

## 7. Conclusions

The widespread overuse and inappropriate assignment of antibiotics has cast a shadow on the possibility of an effective *H. pylori* eradication. While chromosomal mutations in drug target sites represent the most explored mechanism of antibiotic resistance of *H. pylori*, other phenomena may constitute an alternative mode generating resistance to antimicrobials. It is believed that one of them is the activity of efflux pumps—proteinaceous cytoplasmic transporters ejecting chemical compounds to the extracellular environment. Strains of *H. pylori* are equipped with many efflux pumps belonging to four superfamilies, such as RND, MFS, ABC and MATE. The best studied include RND systems, while MATE proteins remain the least explored. An additional group not classically involved in this categorization are metabolic transporters, although their importance in stress responses of *H. pylori* should not be underestimated. As shown by a current detailed literature review, both efflux pumps and metabolic transporters play an important role in the removal of various chemically unrelated classes of antimicrobial compounds (antibiotics, fluorescent dyes, bile salts and heavy metal ions) and take part in the generation of resistance against them. As proved in different studies, an overexpression of genes encoding efflux pumps promotes not only the MDR phenotype, but also intensifies biofilm formation of *H. pylori*. It is noteworthy that, for some efflux pumps of this bacterium, other functions have been discovered. These include the bacterial motility, the activity of urease, the lipopolysaccharide biogenesis, and the maintenance of the energy balance. For this reason, it seems reasonable to consider efflux pump inhibitors as an interesting new mode to fight antibiotic-resistant *H. pylori* strains in the future.

## Figures and Tables

**Table 1:** Point mutations responsible for antibiotic resistance of *Helicobacter pylori* [11,13].

Antibiotic	Antibiotic Class	Molecular Target	Typical Mechanism of Resistance
Amoxicillin	Beta-lactams	Cell wall synthesis	Mutations in *pbp1a*, *pbp2* or *pbp3*
Clarithromycin	Macrolides	Protein synthesis	Mutations in *23S rRNA*
Tetracycline	Tetracyclines	Protein synthesis	Mutations in *16S rRNA*
Rifabutin	Ansamycins	DNA transcription	Mutations in *rpoB*
Levofloxacin	Fluoroquinolones	DNA replication	Mutations in *gyrA* or *gyrB*
Metronidazole	Nitroimidazoles	DNA structure	Inactivation of *rdxA* and/or *frxA*

**Table 3:** Metabolic transporters of *Helicobacter pylori* and their involvement in antibiotic resistance.

Metabolic Transporter	Metabolism-Related Substrates	Antibiotic Substrates	Association with MDR	References
HP0471 (KefB)	K^+^ ions	penicillin G, ampicillin, amoxicillin, tetracycline, clarithromycin, ciprofloxacin, metronidazole, furazolidone	+	[63,64]
HP0497	Na^+^ and Cl^−^ ions			
HP0939 (YckJ)	Amino acids			
HP1017 (RocE)	Amino acids	clarithromycin	*ND*	[63]

Abbreviations: MDR, multidrug resistant; *ND*, no data.
